# The Intercellular Metabolic Interplay between Tumor and Immune Cells

**DOI:** 10.3389/fimmu.2014.00358

**Published:** 2014-07-28

**Authors:** Tingting Wang, Guangwei Liu, Ruoning Wang

**Affiliations:** ^1^Center for Childhood Cancer and Blood Disease, The Research Institute at Nationwide Children’s Hospital, Columbus, OH, USA; ^2^Key Laboratory of Medical Molecular Virology of Ministries of Education and Health, Department of Immunology, School of Basic Medical Sciences, Fudan University, Shanghai, China; ^3^Biotherapy Research Center, Fudan University, Shanghai, China; ^4^Hematology/Oncology & BMT, The Research Institute at Nationwide Children’s Hospital, Columbus, OH, USA; ^5^Department of Pediatrics, The Ohio State University School of Medicine, Columbus, OH, USA

**Keywords:** metabolism, tumor, tumor immunity, antagonism, symbiosis

## Abstract

Functional and effective immune response requires a metabolic rewiring of immune cells to meet their energetic and anabolic demands. Beyond this, the availability of extracellular and intracellular metabolites may serve as metabolic signals interconnecting with cellular signaling events to influence cellular fate and immunological function. As such, tumor microenvironment represents a dramatic example of metabolic derangement, where the highly metabolic demanding tumor cells may compromise the function of some immune cells by competing nutrients (a form of intercellular competition), meanwhile may support the function of other immune cells by forming a metabolic symbiosis (a form of intercellular collaboration). It has been well known that tumor cells harness immune system through information exchanges that are largely attributed to soluble protein factors and intercellular junctions. In this review, we will discuss recent advance on tumor metabolism and immune metabolism, as well as provide examples of metabolic communications between tumor cells and immune system, which may represent a novel mechanism of conveying tumor-immune privilege.

## Intercellular Metabolic Interaction

The intercellular communication largely relies on the information exchange via soluble factors (e.g., proteins and microRNAs) and direct cell–cell contacts. Beyond this, the shuttling of metabolites may serve as an additional form of intercellular communication and a high degree of intercellular coordination in various physio-pathological situations. As an intensive energy-consuming organ, brain efficiently utilizes nutrients/metabolites via organizing a complex intercellular shuttle of glucose, glutamine, glutamate, pyruvate, and lactate between neurons and astrocytes (1). Similar metabolic coordination exists in retina between glial cells and photoreceptor cells, in muscle between fast white fibers and slow red fibers, and in testis between sertoli cells and spermatogenic cells (2, 3). Also, emerging evidences have shown that various pathogen-derived metabolites mediate an intercellular host–pathogen interaction and critically impact on homeostasis and pathogenesis during pathogen invasion (4–7). Tumor microenvironment represents a dramatic example of metabolic derangement, where tumor-surrounding cells may either compromise or support highly metabolic demanding tumor cells by competing nutrients (a form of intercellular competition) or by forming a metabolic symbiosis (a form of intercellular collaboration), respectively. Amino acids, lactate, and lipids derived from stromal cells, adipocytes, mesenchymal stem cells, epithelial cells, or tumor cells from hypoxic regions can modulate tumor cell growth and their responses to therapy (8–15). Beyond this, the immune system, a pivotal cellular compartment presented in tumor microenvironment, is intimately involved in tumor initiation, progression, and responses to therapy.

## Tumor Immunity

Interaction of immune system with tumor is a complex and dynamic process. As the major component of anti-tumor immunity, tumor antigen-specific cytotoxic T (CTL) and T effector (T_eff_) cells together with antibody-producing B cells and antigen-presenting dendritic cells (DC) elicit adaptive anti-tumor activity through direct recognizing and killing tumor cells and orchestrating a plethora of adaptive and innate immune responses. Also, macrophages, natural killer (NK) cells, and NK-T cells form an important layer of non-specific innate immunity to suppress tumor progression. However, tumor often co-opts and manipulates its microenvironment favoring the development of immunosuppressive cells, such as myeloid-derived suppressor cells (MDSC) and regulatory T (T_reg_) cells. In addition, tumor-associated macrophages (TAMs), a pivotal immune population within the tumor microenvironment, are composed of multiple distinct pro- and anti-tumoral subpopulations. Mounting evidence indicates that strengthening the amplitude and quality of T cell-mediated adaptive response is one of the most promising approaches to enhance therapeutic anti-tumor immunity (16–19).

## Metabolic Reprograming in Tumor

The shift from glucose oxidation toward aerobic glycolysis, also termed “Warburg effect,” and heightened glutamine catabolism are characteristic hallmarks of cancer cells. The metabolic rewiring of cancer cells supporting tumor growth and survival relies on a hierarchical oncogenic cascade involved in Akt/mTOR, MAPK, and essential transcriptional factors, such as HIF1α- and Myc-dependent metabolic transcriptome. Secretion of metabolic end products, such as lactate from glycolysis and glutaminolysis and CO_2_ from the pentose phosphate pathway, often result in an extracellular acidification in tumor microenvironment. Hence, the Na^+^/H^+^ exchanger, the H^+^-lactate co-transporter, monocarboxylate transporters, and the proton pump (H^+^-ATPase) that are frequently activated in cancer cells play essential role in modulating the pH and ionic compositions in tumor microenvironment (20–22). Also, elevated expression of phosphoglycerate dehydrogenase (PHGDH) and it-mediated diversion of glycolysis into serine biosynthetic pathway have been found to be essential for the proliferation of certain tumor cells, such as breast cancer cells and melanoma cells (23, 24). Beyond being key precursors for biosynthesis, metabolic products of tryptophan, cysteine, serine, and glycine also contribute to tumor homeostasis through modulating anti-oxidative response and anti-tumor immunity (8, 25–28). Together, the metabolic reprograming in tumor contributes to its growth either by directly supporting cancer cell proliferation or by shaping the microenvironment potentially favoring tumor cell survival.

## Metabolic Reprograming in Immune System

Recent studies from us and others have indicated that metabolic regulation and cell signaling are tightly and ubiquitously linked with immune responses (29–34). The distinct metabolic profiles of lymphocytes are intimately linked to their status and function (Table 1). Naïve T lymphocytes rely mainly on fatty acid oxidation and some glycolysis to fulfill their energy demand for survival. Upon stimulation, activated T lymphocyte “reprograms” its metabolism, by dramatically increasing aerobic glycolysis and glutaminolysis meanwhile decreasing lipid oxidation to meet its requirements for cell size growth, cell division, and cytokine production (35–38). In contrast, stimulation of B lymphocytes leads to a balanced increase in aerobic glycolysis and oxygen consumption (39, 40). As T lymphocytes begin to proliferate, they also undergo differentiation into functional subsets in response to extracellular signals, and these subsets determine the nature of the immune response. According to the nature of initial antigen challenge and specific cytokine signals, activated CD4 T cells differentiate into T_eff_, including T helper Th1, Th2, Th9, and Th17, follicular helper Tfh, and T_reg_. Th1 cells mediate responses to intracellular pathogens. Th2 cells control responses to extracellular bacteria and helminthes. Th9 cells play a role in the pathogenesis of asthma and resolution of parasitic infections. Th17 cells are important in anti-fungal defense and inflammation. Tfh cells are the specialized B cell helper. T_reg_ cells dampen immune responses by suppressing T cell activation and inflammatory response. The predominant metabolic program in T_reg_ cells is mitochondrial-dependent oxidation of lipid and potentially other mitochondrial-dependent metabolites. It has been indicated that forcing proliferating T cells to utilize free fatty acids for energy tends to drive enhanced T_reg_ differentiation (41). In contrast, increased aerobic glycolysis is seen in Th1, Th2, and Th17 cells, and partially due to activation of PI3K/Akt/mTOR pathway. The transcription factor HIF1 (hypoxia-inducible factor 1) has also been characterized as a key regulator of the anabolic metabolism in differentiating Th17 cells (42, 43). Similar to CD4 T cells, CD8 T cells also switch from fatty acid oxidation to aerobic glycolysis upon activation. The glycolysis and anabolic metabolism are essential for CD8 T cell growth and differentiation into cytotoxic T cells (34). After the peak of the primary T cell response, the metabolic state in CD8 T cells shifts from glycolysis back to lipid oxidation, which is pivotal for cell survival and the generation of CD8 T memory cells (44, 45).

**Table 1:** **Metabolic profiles of immune cells**.

Immune cells	Naïve T cells	Activated T cells	T_eff_ cells	T_reg_/T memory cells	Activated dendritic cells	M1 macrophages	M2 macrophages (TAM)
Metabolic profile	fatty acid oxidation and some glycolysis	Glycolysis and glutaminolysis	Glycolysis	Fatty acid oxidation	Glycolysis	Glycolysis, pentose phosphate shunt (PPP), glutamine, and arginine catabolism	Lipid oxidation

Dendritic cells and macrophages are first-line effectors of innate immunity. DC maturation is concomitant with a metabolic switch to aerobic glycolysis (46, 47). Aerobic glycolysis fulfills bioenergetic need and also provides building blocks for the biosynthesis of macromolecules, such as lipids, a proper balance between uptake and synthesis of which is required for immunogenicity of DCs (48, 49). As functionally plastic cells, macrophages are capable of tightly coordinating their metabolic programs with their functional properties. This allows macrophages to grow, survive, and properly respond to a variety of pathophysiological signals in their changing microenvironments. Within the tumor microenvironment, TAMs are often identified as pro-tumoral M2 type macrophage (50, 51). Mounting evidences have showed that switching the TAM phenotype from M2 to M1 may promote anti-tumor activity, implicating a phenotypic plasticity of TAM (52–57). To mount a rapid inflammatory response, M1 macrophages coordinately engage aerobic glycolysis, pentose phosphate shunt (PPP), glutamine, and arginine catabolism to produce nitric oxide (NO) and reactive oxygen species (ROS) (58–60). However, anti-inflammatory M2 macrophages largely utilize lipid oxidation (61–63) meanwhile shift arginine catabolism from iNOS-mediated production of NO to the production of urea and ornithine (64–68). Similar to macrophage, the polarization of MDSC, a heterogeneous immunosuppressive population in tumor microenvironment, toward a pro-inflammatory phenotype (often referred as M1) is associated with heightened glycolysis meanwhile reduced immunosuppressive function (69). Beyond this, metabolic regulation in NK and neutrophils are largely unknown.

## Metabolic Antagonism and Symbiosis in Tumor Microenvironment

**Figure 1:**
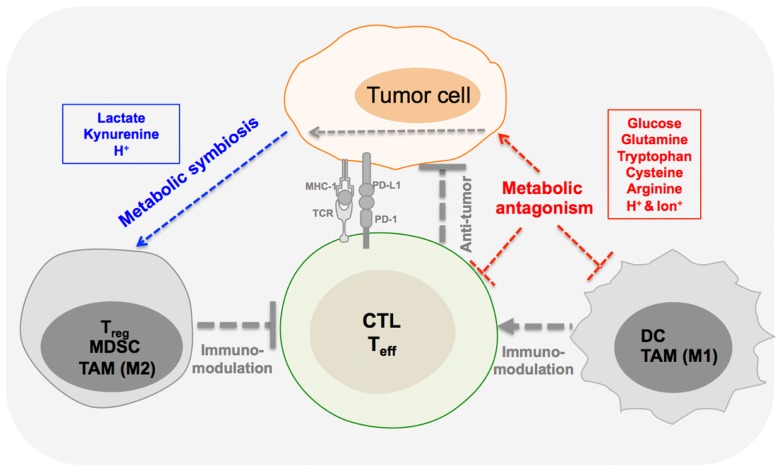
**Metabolic interplay in tumor immunity**. Metabolic interplay through shuttling of metabolites among different cell compartments in tumor microenvironment serves as a form of intercellular communication and intercellular coordination. T effector cells (T_eff_), cytotoxic T cells (CTL), dendritic cells (DC), and tumor-associated macrophage (TAM-M1) may form a potential metabolic antagonism (red color marked) with tumor cells. On the contrary, T regulatory cells (T_reg_), myeloid-derived suppressor cells (MDSC), and TAM-M2 may preferentially utilize metabolic products of tumor to form a potential metabolic symbiosis (blue color marked) in tumor microenvironment.

Aerobic glycolysis and glutaminolysis are dominant cancer metabolic routes. Heightened glucose and glutamine consumption often results in the depletion of nutrients (glucose, glutamine, etc.) whereas accumulates metabolic end- or by-products (lactate, proton, etc.) in tumor microenvironment (70, 71). In addition to the above general metabolic features that are required to support the needs of proliferation and other neoplastic features, tumor cells also exhibit diverse metabolic phenotypes that are often due to the adaptation of pre-existing cell/tissue lineage specific metabolic network. It is well documented that in tumor cells, biosynthesis, and catabolism of glycine and serine, as well as catabolism of tryptophan and cysteine, are essential to support tumor cell survival (25, 72–74). Acidic extracellular pH, which is resulted from the accumulation of lactate and CO_2_ production, has been demonstrated to be important for cancer progression (75, 76). Recent studies have demonstrated that anti-tumoral immune population, such as CTL and T_eff_ cells, engage robust aerobic glycolysis and glutaminolysis, suggesting a potential metabolic antagonism (competition) for nutrients between tumor and those immune cells. On the contrary, pro-tumoral immune suppressive cells may preferentially utilize metabolic products of tumor to form a potential metabolic symbiosis in tumor microenvironment (Figure 1).

### Antagonism

#### Glucose and glutamine

The similarity of metabolic programs between tumor and T cells (CTL and T_eff_) leads to fierce competition for limited source of glucose and glutamine in local environment. The restriction of glucose and glutamine to fast proliferating cells could result in metabolic stress on both tumor and immune cells. Nutrient deprivation elicits signaling responses through AMP-dependent kinase (AMPK), mammalian target of rapamycin (mTOR), transcriptional factor p53, and other unknown signaling modulators to confer metabolic plasticity allowing cancer cells survive under low glucose and low glutamine condition (77–81). Furthermore, lactate and CO_2_ produced from glycolysis and glutaminolysis lead to microenvironment acidification, favoring the development of more aggressive and invasive tumor cells (75, 76). Unlike tumor cells, metabolic stresses are less tolerated in non-malignant cells, such as T cells, and are often immune suppressive, partially due to a preferential development of T_reg_ cells following nutrients restriction (41, 42). Several studies also indicated that nutrient starvation perturbs T_eff_ cytokine production, macrophage phagocytic activity, and superoxide production. Therefore, metabolic microenvironment may render tumor cells a selective advantage due to their resistance to apoptosis and rapid adaptation under metabolic stress.

#### Tryptophan catabolism

The catabolism of the essential amino acid tryptophan has been reported to be a biomarker of tumor tissues in various studies. In tumor cells, the conversion of tryptophan to kynurenine is primary mediated by two dioxygenases, indoleamine-2,3-dioxygenase (IDO) and tryptophan-2,3-dioxygenase (TDO). IDO is expressed in many types of tumor cells and antigen-presenting cells, whereas TDO exists in certain IDO-negative tumor cells, such as malignant gliomas and hepatocellular carcinoma (82–85). The breakdown of tryptophan has been shown to dramatically affect the function of T cells against tumor cells. On one hand, upregulated tryptophan catabolism in tumor tissue resulted in the depletion of tryptophan in the extracellular space, which causes T_eff_ cells anergy and apoptosis and, in turn, suppresses anti-tumor-immune responses. On the other hand, kynurenine and potentially other catabolic metabolites of tryptophan are nature ligands of aryl hydrocarbon receptor (AHR), which plays a broad role in modulating immunity (86, 87). As such, extracellular accumulation of kynurenine elicits an AHR-mediated response to reciprocally enhance function of T_reg_ and suppress function of T_eff_ and immunogenicity of DCs (88–90). Thus, tryptophan depletion and kynurenine accumulation cooperatively create an immunosuppressive microenvironment in tumor (25, 90).

#### Cysteine and glycine

Physiological levels of ROS play essential roles in various signaling cascades for cell survival and proliferation, whereas excess ROS causes cell injury and tissue damage (91, 92). The thiol group in glutathione (GSH) acts as a reducing agent that can quench the cytotoxic ROS, and thus GSH is considered as an essential cellular antioxidant system to maintain redox homeostasis. Heightened GSH level is observed in numerous types of cancers, and the enriched GSH improves tumor cell survival by protecting them against oxidative stress (73, 74). Tumor cells uptake cysteine and cystine from the local environment and convert them into GSH together with glutamate and glycine, which are often derived from glutamine and glucose. Similarly, T cell proliferation depends on the uptake of exogenous cysteine. T cells lack cystathionase enzyme that converts methionine to cysteine and xc-transporter that imports cystine as an alternative source of cysteine (93). Thus, the competition between tumor cells and T cells for cysteine and glycine may lead to the suppression of T cell activation and proliferation.

#### Arginine catabolism

As another form of free radical, NO plays multifaceted roles in cancer initiation, progression, differentiation, and angiogenesis (94–96). In mammals, NO is converted by a family of nitric oxide synthases (NOS) from arginine. It has been reported that arginine depletion retards the growth of some types of tumor, whereas other studies demonstrated that arginine supplementation assists anti-tumor treatment possibly by enhancing immune function (97, 98). As such, arginine has been discovered to stimulate T cell and NK cell activity and promote production of pro-inflammatory cytokines (99, 100). Also, tumor-derived NO may elicit cytotoxic effects on tumor-associated immune cells. However, the intrinsic resistance to NO-mediated cytotoxicity of tumor cells with mutated p53 offers a selective growth advantage of cancer cells over normal cells (101–104).

#### Proton and sodium ion

It has been known that the acidification of microenvironment caused by the accumulation of lactic acid and CO_2_ enhances tumor radioresistance and favors tumor cell migration and invasion (75, 76, 105). Beyond this, acidic environment decreases the activity of NK cells, suppresses T cell proliferation, and impairs cytokine production and cytotoxic activity of T cells. Accumulating evidences also suggest that acidic microenvironment has a profound impact on monocytes differentiation and cytokine release (106–108). As one of the key inorganic ions in our body, cross-membrane transport of sodium ion is intimately coupled with proton and amino acids transport and also profoundly impact on tumor microenvironment (75, 109). Recent studies show that high-sodium chloride conditions induce the development of pathogenic Th17 cells with elevated release of pro-inflammatory cytokines (GM-CSF, TNF-α, and IL-2) and thus promote tissue inflammation, which may either promote or suppress tumor formation. While some of the effects of sodium are mediated through serum/glucocorticoid-regulated kinase 1(SGK1), further investigations are warranted to assess the impact of sodium on proton and amino acids transport. As such, a sodium ion-proton axis may coordinately modulate anti-tumor response (110, 111).

### Metabolic symbiosis

In contrast to T_eff_ cells, the enriched lactate and the acidic microenvironment do not have obviously suppressive effect on T_reg_ cells, as they have a different energy metabolism that relies on fatty acid oxidation. The lactate accumulated in the microenvironment is generally considered as metabolic “waste.” However, numerous studies have indicated the possible function of lactate as a prominent substrate in oxidative metabolism among various types of cells such as muscle cells, neurons, and certain tumor cells (112–114). Although, it has not been demonstrated, the preference of mitochondrial-dependent oxidative metabolism of T_reg_ indicates the possibility that T_reg_ may utilize lactate under nutrient scarcity, which often happens in tumor microenvironment. The concentration of lactate in vertebrate plasma ranges from 1 to 30 mM under physiological and pathological conditions (2). Beyond serving as a potential alternative energy source, early studies suggested that high lactate concentrations (2–30 mM) enhance T_reg_ differentiation through the stimulation of IL-2 production (115, 116). Similarly, increased production of lactate by tumor cells promotes the development of MDSC (117). Also, lactate and acidic environment have a profound impact on secretory profile of TAM, promoting tumor angiogenesis (108, 118, 119). Beyond lactate, the catabolic metabolites of tryptophan, such as kynurenine, promote T_reg_ differentiation and immune suppressive function (89). Thus, tumor-derived lactate and tryptophan catabolic metabolites may form a layer of metabolic symbiosis with various immune cells to favor tumor growth.

## Conclusion and Perspective

The metabolites that present in tumor microenvironment may also have signaling functions independent of their roles of bioenergetics fuels. This may represent a general feature of the intercellular metabolic crosstalk mediated by metabolites. To fully understand the underlying complexity of intercellular metabolic interplay, new techniques that allow us to quantitative measure metabolites, assess metabolic flux *in situ*, and detect physical interaction between metabolites and cell surface proteins are warranted. The fast moving cancer metabolism field and immunotherapy field have generated tremendous excitement regarding new therapeutic strategies and will likely change the paradigm of therapeutic interventions for cancer. However, the perturbed metabolic landscape of the tumor microenvironment can have a profound impact on anti-tumor immunity. As such, understanding the metabolic interplay between tumor and immune system will guide the development of optimal metabolic interventions on cancer without compromising anti-tumor immunity. Beyond this, intercellular metabolic interplay may also play an essential role in forming a pro-tumoral inflammatory microenvironment.

## Conflict of Interest Statement

The authors declare that the research was conducted in the absence of any commercial or financial relationships that could be construed as a potential conflict of interest.
